# Circulating endothelial cells are an early predictor in renal cell carcinoma for tumor response to sunitinib

**DOI:** 10.1186/1471-2407-10-695

**Published:** 2010-12-31

**Authors:** Viktor Gruenwald, Gernot Beutel, Susanne Schuch-Jantsch, Christoph Reuter, Philipp Ivanyi, Arnold Ganser, Marion Haubitz

**Affiliations:** 1 Hannover Medical School, Carl-Neuberg-Strasse 1, 30625 Hannover, Germany Clinic for Hematology, Hemostasis, Oncology and Stem Cell transplantation; 2 Hannover Medical School, Carl-Neuberg-Strasse 1, 30625 Hannover, Germany Department of Nephrology

## Abstract

**Background:**

Tyrosine kinase inhibitors (TKI) have enriched the therapeutic options in patients with renal cell carcinoma (RCC), which frequently induce morphological changes in tumors. However, only little is known about the biological activity of TKI. Circulating endothelial cells (CEC) have been associated with endothelial damage and, hence, may serve as a putative marker for the biological activity of TKI. The main objective of our study was to evaluate the predictive value of CEC, monocytes, and soluble vascular endothelial growth factor receptor (sVEGFR)-2 in RCC patients receiving sunitinib treatment.

**Methods:**

Analyses of CEC, monocytes, and sVEGFR-2 were accomplished for twenty-six consecutive patients with metastatic RCC who received treatment with sunitinib (50 mg, 4 wks on 2 wks off schedule) at our institution in 2005 and 2006.

**Results:**

In RCC patients CEC are elevated to 49 ± 44/ml (control 8 ± 8/ml; P = 0.0001). Treatment with sunitinib is associated with an increase in CEC within 28 days of treatment in patients with a Progression free survival (PFS) above the median to 111 ± 61 (P = 0.0109), whereas changes in patients with a PFS below the median remain insignificant 69 ± 61/ml (P = 0.1848). Monocytes and sVEGFR2 are frequently altered upon sunitinib treatment, but fail to correlate with clinical response, defined by PFS above or below the median.

**Conclusions:**

Sunitinib treatment is associated with an early increase of CEC in responding patients, suggesting superior endothelial cell damage in these patients as a putative predictive biomarker.

## Background

Tyrosine kinase inhibitors (TKI) were recently successfully added to the armentarium to treat renal cell carcinoma (RCC). Sunitinib, a first generation TKI which targets VEGFR1-3, PDGFR α/ß, KIT, RET, CSF 1R and FLT-3, has recently been approved for the treatment of RCC [1]. Its antitumor activity is at least partially mediated through inhibition of tumor vessel formation, which can be demonstrated through sophisticated imaging techniques, such as dynamic contrast enhanced MRI. As these techniques are not commonly available to most physicians, biomarkers which predict biological and antitumor activity are desperately needed to adequately monitor tumor therapy and predict tumor response to sunitinib.

In RCC, inhibition of vessel formation is thought to be the prime mechanism to achieve antitumor activity [2]. The biological relevance of the different VEGFR family members in this process was elucidated in murine models, and VEGFR-2 was determined to be the main regulator of neo-angiogenesis and the most promising target for therapeutic intervention [3]. Various activating ligands were identified, which may bind with a distinct affinity to VEGFR family members. Inhibition of these targets correlated with significant changes of circulating proteins and the application of sunitinib was associated with changes of circulating VEGF, placental growth factor (PlGF) and sVEGFR-2 [4-6]. So far, such changes were associated with target inhibition *in vivo *but failed to predict tumor response in patients [4,7].

Other markers, such as circulating endothelial cells (CEC), have been studied in order to define the biological response to these agents. Increased CEC levels were demonstrated to correlate with vascular damage and are observed in a variety of vascular disorders [8-11]. CEC were thought to be shed from the endothelium and successfully predict the activity of vessel damage seen in vasculitis [11]. In cancer patients, elevated CEC levels were also detected [12] and apoptotic CEC were recently proposed to predict clinical outcome of metronomic therapy in breast cancer patients [13]. In addition, the predictive value of soluble markers was studied in treatments with angiogenesis inhibitors. Soluble VEGFR-2 levels were reported to decrease during sunitinib treatment but were not predictive for response in RCC and GIST patients [4,5].

In this pilot study, we investigated the role of CEC and sVEGFR2 as potential biomarkers in metastatic RCC patients who were treated with sunitinib. Blood samples were collected prior to and during the course of sunitinib therapy and tumor response was monitored according to RECIST criteria. Biomarkers were analyzed for responding and non-responding patients either for kinetic changes during the course of treatment or as a single predictive marker prior to drug-exposure.

## Methods

### Patients

The study was conducted in accordance with the Declaration of Helsinki and the local Institutional Review Board approved the study protocol. Informed consent was obtained prior to blood collections. 26 patients with metastatic RCC were included in the analyses (Table 1).

**Table 1:** Patients' Characteristics

	No. of patients	(%)
**ECOG**		
**0**	24	92
**1**	2	8
**Nephrectomy**	24	92
**Histology**		
Papillary	2	8
Sarcomatoid	2	8
Chromophobe	1	4
clear cell	21	80
**Age (range)**	62 (45-80) years	
**Male**	15	
**Female**	11	
**Median PFS in days (range)**	249 (63-953)	
**Best response**		
Objective response (OR)	11	42
Stable disease (SD)	9	35
Progressive disease (PD)	6	23

Best response to therapy was defined as either stable disease (SD) or objective response (OR) according to RECIST criteria, and was determined by CT-scans at baseline and every other cycle. Due to limited sample size, responders were defined by either SD or OR, and patients with progressive disease (PD) were deemed non-responders. A total of 6 non-responders and 20 responders were identified within the study population. No patient received treatment with a VEGFR-inhibitor prior to sunitinib. 15 male and 11 female patients entered the study with a mean age of 62 years (range 45-80). 18 patients had received at least one prior regimen and 8 patients were treatment naïve. Blood samples from 20 healthy volunteers with a mean age of 59 (range 45-77) served as normal controls.

### Study design

Blood samples for analyses of CEC, monocytes and sVEGFR2 were collected in parallel in patients who received treatment with sunitinib (50 mg OD 4 weeks on - 2 weeks off) for metastatic RCC as standard of care treatment prior to start of treatment, at day 14, day 28 and with the last dose of sunitinib of each subsequent course for the duration of sunitinib therapy. Monocytes were detected according to institutional standard on day 1, 14 and 28 during the first cycle and day 1 and/or 28 during subsequent cycles by complete blood counts. The study was performed without external financial support.

### Preparation of blood samples

Blood samples were collected from 26 patients. Available probes at baseline consisted of 26 for CEC, 26 for monocytes and 18 for sVEGFR2 analyses. After 14 days of treatment, 17 probes were assessed for CEC, 23 for monocytes and 14 for sVEGFR2. After 28 days, a total of 23 probes were analyzed for CEC, 22 for monocytes and 17 for sVEGFR2.

Blood samples for CEC analyses were taken and analyzed as previously described [14]. In brief, samples were analyzed within 4 hours of non-traumatic venous puncture in 7.5 ml ethylene-diamine tetra-acetic acid and stored at 4°C if readout was not performed immediately. Anti-CD 146-coated M-450 Dynabeads (Dynal, Norway) were prepared as described by the manufacturer. 1 ml blood was mixed with 1 ml buffer (phosphate-buffered saline, 0.1% bovine serum albumin, 0.1% sodium azide, and 0.6% sodium citrate) on ice, supplemented by 20 μl of FcR-blocking agent (Miltenyi, Bergisch Gladbach, Germany) and 50 μl anti-CD 146-coated Dynabeads (10 μg ml^-1^). Samples were mixed (30 min.) and rinsed with buffer inside the magnet at 4°C and stained with FITC-coupled Ulex europaeus lectin-1 solution (UEA-1, Sigma-Aldrich, St. Louis, USA) for 1 hour in darkness. CEC were then washed and visualized by fluorescence microscopy at 553 nm, and counted employing a Nageotte chamber. Serum for sVEGFR2 was immediately stored at -20°C and analyzed by ELISA-tests according to manufacturer's description (R&D Systems, Minneapolis, USA).

### Statistical analyses

Paired and unpaired Student's t-Test were employed for statistical analyses. Paired Student's t-Test has been utilized in groups with repeated measurements all other t-test evaluations consisted of unpaired t-Tests. Kaplan-Meier estimates were used to plot median progression free survival (PFS). Descriptive statistics are utilized to show the fraction of patients with distinct variables. Standard deviation is reported, if not otherwise specified.

## Results

### Patients with metastatic RCC exhibit higher CEC baseline levels than normal controls

**Figure 1:**
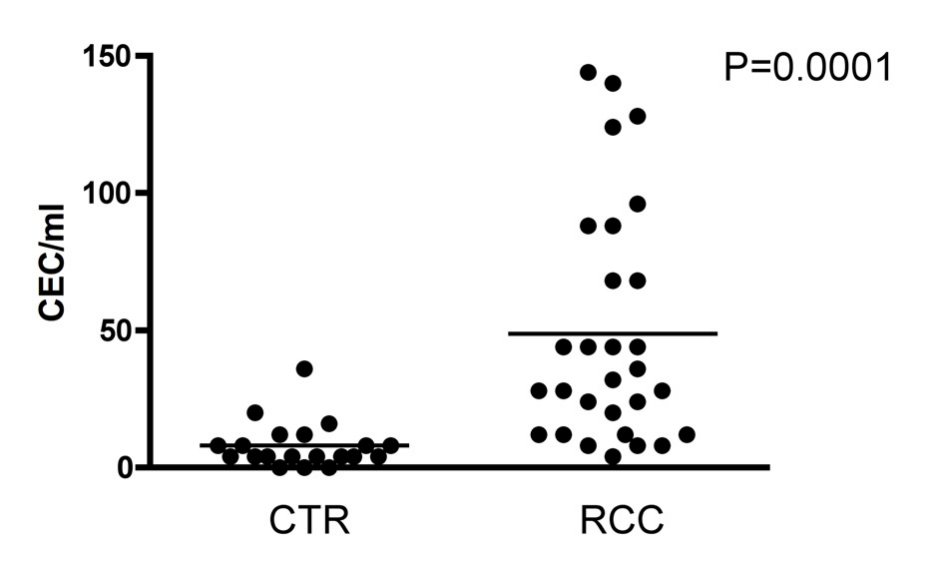
**Levels of CEC are elevated in metastatic RCC patients**. Baseline levels of CEC in metastatic RCC patients were significantly elevated compared to normal controls (CTR) (P = 0.0001). Mean values for CEC were 8 ± 8 and 49 ± 44/ml in CTR and RCC patients, respectively.

A total of 26 patients with metastatic RCC were included in our study. Patients' characteristics are depicted in table 1. In order to evaluate the role of CEC in RCC, we first evaluated values in normal controls and untreated RCC patients. Mean CEC values in metastatic RCC patients were significantly higher than those from normal controls (49 ± 44 CEC/ml vs. 8 ± 8 CEC/ml; P = 0.0001) (Figure 1).

### Treatment with sunitinib is associated with an increase of CEC as an early biological response

**Figure 2:**
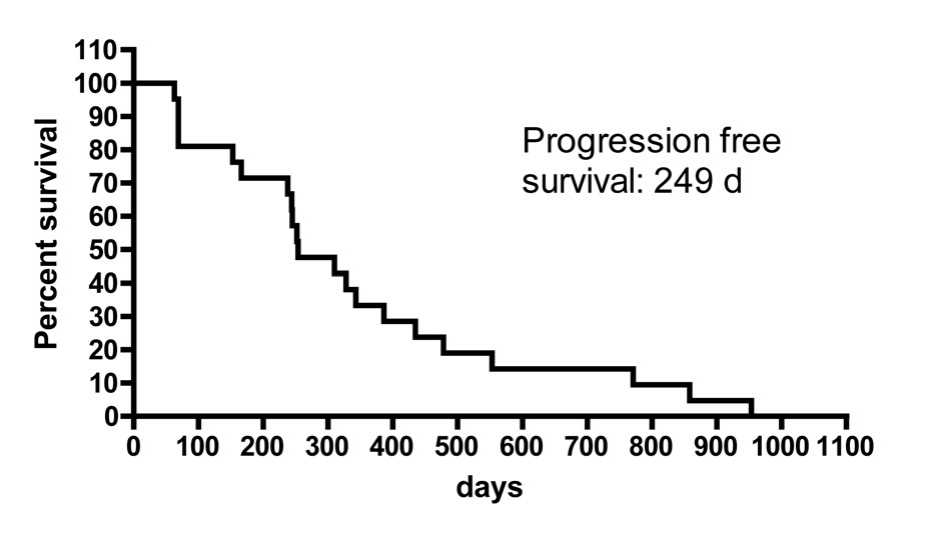
**Progression free survival of sunitinib treatment**. The median progression free survival (PFS) with sunitinib is shown by Kaplan-Meier estimate (median 249 days).

Sunitinib achieved a median PFS of 249 days (Figure 2), which was associated with response to therapy (OR and SD) in 77% of the patients. Clear cell histology (N = 21) was the predominant tumor type and response to therapy was noted in 17 patients (81%) and median PFS was 254 days. This subgroup of patients achieved baseline CEC counts of 47 ± 43/ml. However, a distinct subtype was noted in 5 patients (sarcomatoid N = 2; papillary N = 2; chromophobe N = 1) and treatment with sunitinib resulted in tumor response (OR or SD) in 3 patients (60%) and was associated with a median PFS of 156 days. A similar CEC count of 47 ± 46/ml was detected in these patients at baseline (P = 0.9737).

**Figure 3:**
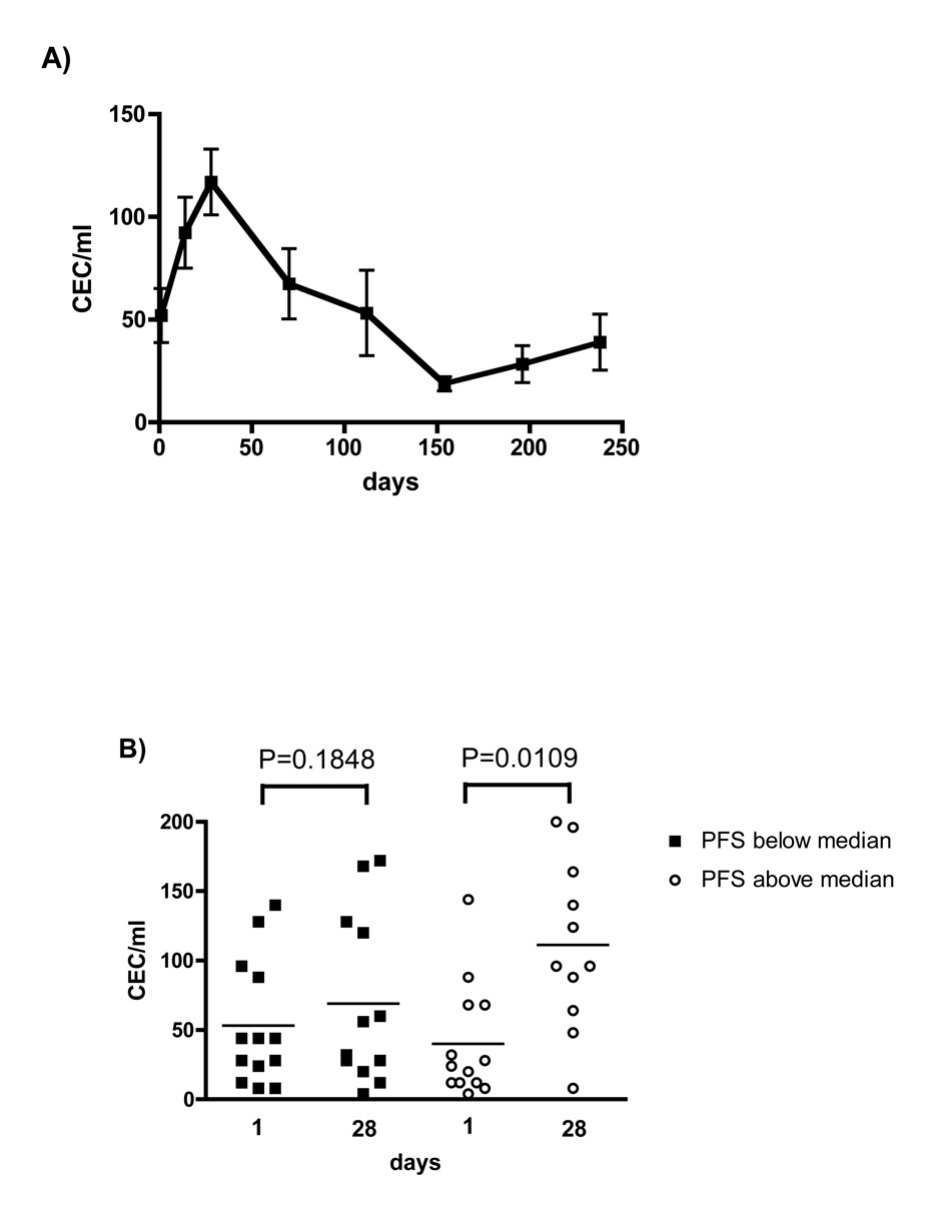
**CEC display a characteristic response to treatment with sunitinib**. A) Characteristic changes of CEC counts are depicted in 13 patients with repeated measures for the duration of 238 days. Curve shows a marked increase within 28 days, followed by decreased CEC counts to a basal level. Bars show mean standard error mean. B) Patients were grouped according to their PFS above or below the median. CEC measured at baseline and after 28 days of treatment with sunitinib are depicted for both groups. In patients with PFS above the median mean CEC values increased significantly from baseline 40 ± 41 CEC/ml to 111 ± 61 (P = 0.0109) at day 28. However, in patients with PFS below the median, the increase from 53 ± 45 to 69 ± 61 CEC/ml remained insignificant (P = 0.1848).

We investigated the kinetics of CEC in patients treated with sunitinib, which is administered over 28 days followed by a 14 day drug holiday. Probes were sampled at baseline, days 14 and 28 during course one and on the last day of drug administration of each course to ensure maximum target inhibition for biological effects. The analyses of all treated patients revealed significant changes after 14 and 28 days of sunitinib treatment. During the first course, mean values increased from 49 ± 44 CEC/ml at baseline to 84 ± 59 CEC/ml after 14 days (P = 0.0331) and 89 ± 63 CEC/ml after 28 days (P = 0.0159) of treatment. CEC declined during the subsequent treatment to baseline levels and below (range 19-58 CEC/ml). The course of CEC during sunitinib treatment is depicted in a subset of patients (N = 13) for whom repeated measures were available for the duration of 238 days (Figure 3A).

To further define whether early CEC increase has a predictive value for sunitinib treatment, we explored CEC changes in subgroups of patients with a PFS above or below the median PFS. An increased number of CEC is thought to be associated with vascular damage, which we considered a favorable early biological response to sunitinib. We therefore analyzed CEC values during the first course of treatment.

Increased CEC numbers were observed in patients who had a PFS above the median. In these patients, mean CEC values increased from 40 ± 41 CEC/ml at baseline to 111 ± 61 CEC/ml at day 28 (P = 0.0109) (Figure 3B). In addition, early assessment at day 14 was performed in 13 patients and showed a similar increase in mean values to 93 ± 63 CEC/ml (P = 0.0067). In contrast, patients who achieved a median PFS below the median exhibited no significant alteration of CEC (53 ± 45 CEC/ml at baseline, 69 ± 61 CEC/ml at day 28; P = 0.1848), supporting the notion that CEC increase may represent a valuable marker for clinical activity (Figure 3B). Early assessment at day 14 was available in 5 patients only. However, in these patients early assessment was associated with an insignificant increase of mean CEC values to 83 ± 30 CEC/ml (P = 0.3659).

While, baseline values of CEC prior to drug exposure showed a trend to lower values (40 ± 41 CEC/ml) in patients with a PFS above the median when compared to patients with a PFS below the median (53 ± 45 CEC/ml), these differences remained insignificant (P = 0.4414) and do not explain treatment outcome as a single pre-dose evaluation.

### Monocyte counts change in response to sunitinib

**Figure 4:**
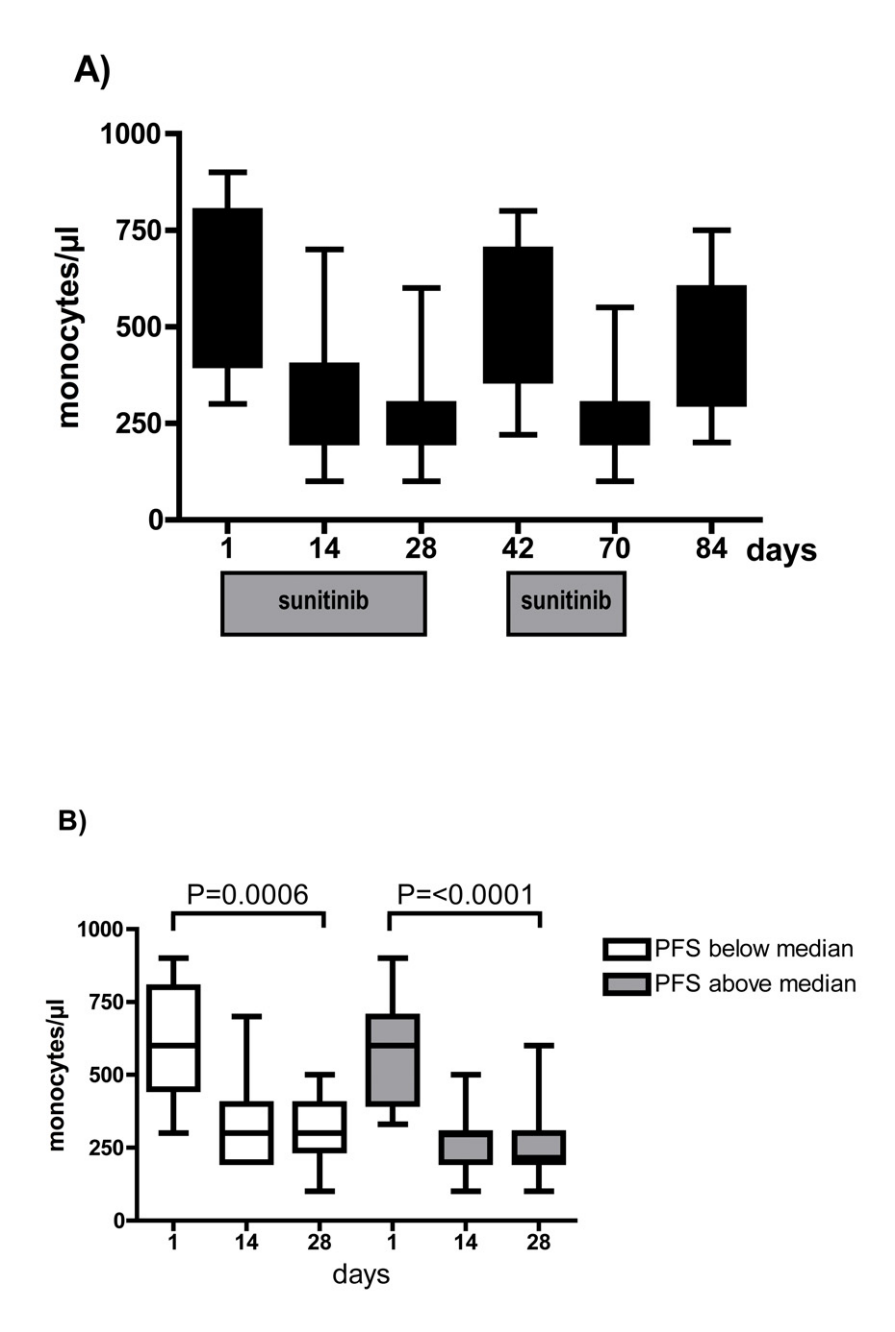
**Treatment with sunitinib decreases circulating monocytes in all treated patients, irrespective of their clinical response to treatment**. A) Monocytes decrease during repeated exposure to sunitinib. However, increased values are measured after the 2-weeks washout interval of sunitinib. B) Patients were grouped according to PFS above or below the median. In both groups, monocyte counts decrease significantly from baseline to day 28 [P = 0.0006 (below median); P < 0.0001 (above median)]. However, monocyte counts at day 1 and 28 remained similar between both groups [P = 0.5701 (day 1), P = 0.3006 (day 28), respectively].

VEGFR-1 promotes vessel growth through recruitment of monocytic blood cells [15], including endothelial progenitor cells (EPC). Monocytes express VEGFR-1 [16] and hence may represent a convenient surrogate marker to measure sunitinib's biological activity. Sunitinib treatment resulted in significant decrease of mean monocyte values from 594 ± 192/μl prior to treatment to 312 ± 136/μl and 278 ± 122/μl at 14 and 28 days of treatment, respectively (Figure 4A). Washout of sunitinib after the first and second treatment course was associated with an increase of monocytes to baseline levels of 501 ± 178/μl and 467 ± 186/μl, respectively, indicating reversible target inhibition. Monocyte response was sustained upon repeated challenge with sunitinib for 28 days (265 ± 108/μl).

All patients showed a similar decline of monocyte counts, irrespective of the duration of PFS to sunitinib. Both groups showed a significant decrease of monocytes on day 28 [P = 0.0006 (below median); P < 0.0001 (above median)]. However, monocyte values at day 14 and 28 were similar in both groups (P = 0.2070, P = 0.3006, respectively) (Figure 4B).

### Treatment with sunitinib is associated with decreased sVEGFR2, but its kinetic change fails to predict clinical response

**Figure 5:**
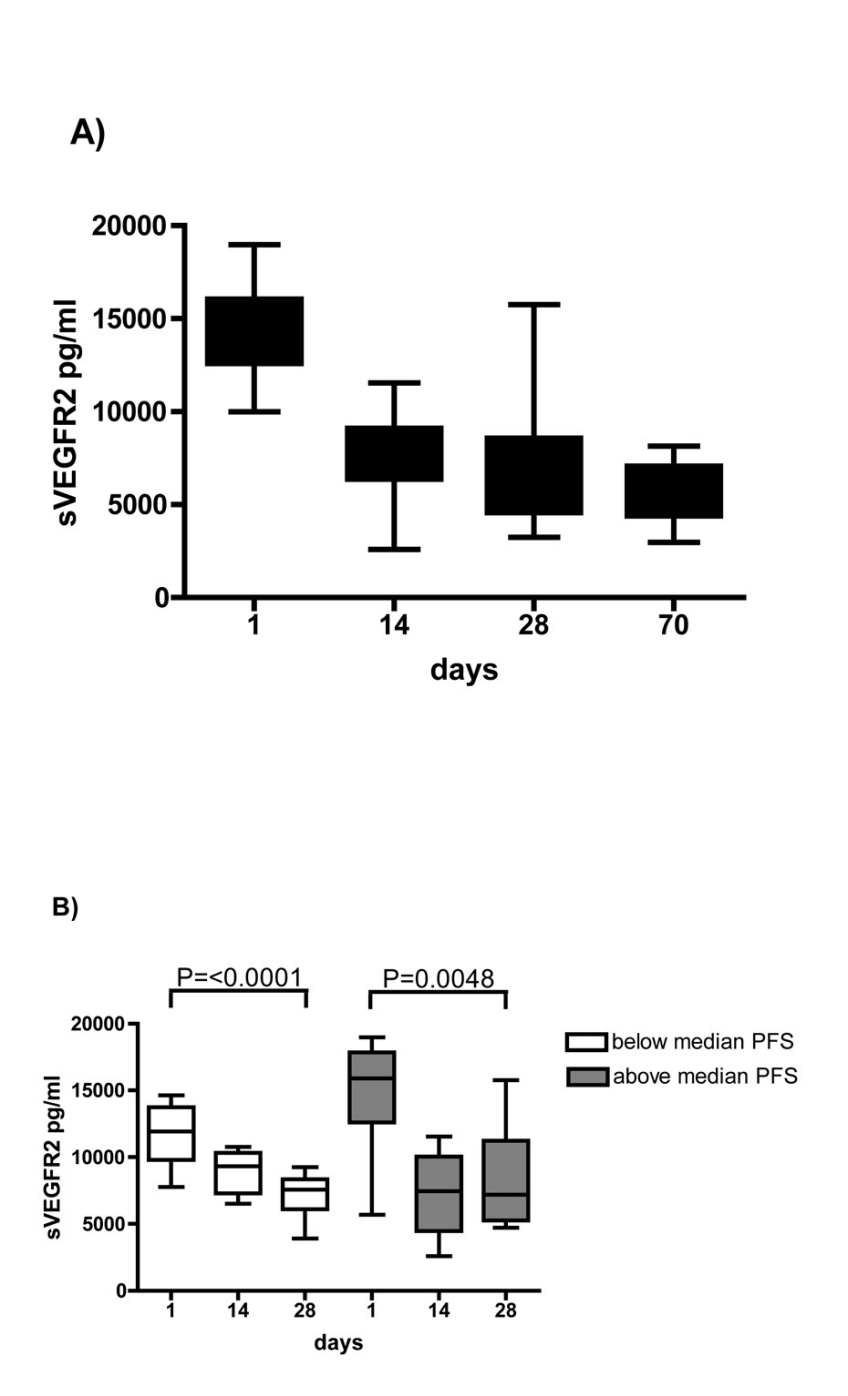
**sVEGFR2 is a sensitive biomarker for sunitinib treatment but fails to predict clinical response**. A) In 16 patients, repeated sVEGFR2 measurements were performed on days 1 - 70. sVEGFR2 declines significantly during treatment with sunitinib (day 28: P < 0.0001). B) Patients were grouped according to PFS above or below the median. Compared to baseline, sVEGFR2 decreased in each group significantly on day 28 [P < 0.0001 (below median), P = 0.0048 (above median), respectively]. However, no difference was shown at day 28 between both groups (P = 3760).

sVEGFR2 was investigated as a potential predictive biomarker for response to sunitinib treatment. The mean sVEGFR2 concentration prior to treatment was 13317 ± 3017 pg/ml. Sunitinib induced a 39% reduction of sVEGFR2 (to 8180 ± 2301 pg/ml; P < 0.0001), which remained repressed throughout further treatment. The pattern of sVEGFR2 response is illustrated in Figure 5A in patients with repeated measurement available for 70 days only. In these patients, sVEGFR2 dropped significantly at day 28 (P < 0.0001) compared to baseline.

We then investigated subgroups of patients according to their PFS to sunitinib, which was either above or below the median. Mean sVEGFR2 levels prior to treatment with sunitinib showed a trend for decreased counts in patients with a PFS above the median (11724 ± 2278 pg/ml) compared to patients with a PFS below the median (14615 ± 4068 pg/ml), however, the difference remained insignificant (P = 0.0814). Both groups showed similar decrease of sVEGFR2 during the latter course of treatment (day 28: 7157 ± 1800 pg/ml and 8484 ± 3737 pg/ml, respectively; P = 0.3760) (Figure 5B).

## Discussion

Targeted agents have recently enriched the treatment of renal cell carcinoma. Objective responses are frequently seen in patients receiving targeted agents and approach 40% for patients treated with sunitinib [1]. However, best objective responses often occur rather late during the course of treatment with VEGFR-inhibitors. Axitinib, a potent inhibitor of VEGFR, showed an objective response in 44% of RCC patients [17], which were registered after 90-403 days of continuous treatment. Two complete responses were detected at days 256 and 362, respectively. Clearly, there is a need to identify biological markers, which allow earlier prediction of tumor response to anti-angiogenesis agents. Numerous markers were recognized to be altered through sunitinib treatment, including VEGFR ligands, soluble receptors and endothelial cells [4-6]. Our work focused on CEC, monocytes and sVEGFR2 in order to elucidate their role as potential predictive markers in RCC patients treated with sunitinib.

Patients with detectable tumors are known to have increased levels of CEC as a consequence of endothelial perturbation, but the implication of this observation for prognosis or tumor response remains unclear [18-20]. Beerepoot et al. reported similar CEC values for healthy controls and patients with SD as best tumor response, whereas patients seemed to have elevated CEC at time of progression [12]. Mancuso et al. reported normal levels of CEC in patients with complete remission of lymphoma [19]. Patients in these studies received conventional chemotherapeutics, which target the tumor rather than its vasculature. Hence, it is conceivable that the role of CEC in anti-angiogenic therapies may be different.

The source of CEC remains mainly undetermined. Chang et al [21] suggested a mutual luminal surface of tumor and endothelial tissue in tumor vessels, which contribute to the continuous shedding of circulating cells into the blood stream. Application of therapeutics which target the tumor vessels is thought to alter the compilation of circulating cells and treatments with angiogenesis inhibitors were associated with changes of CEC and EPC in tumor-bearing mice. ZD6126, an inhibitor of VEGFR2, was associated with a dose-dependent increase of CEC and decrease of microvessel density, suggesting that inhibition of angiogenesis is associated with gain of CEC shedding from the tumor vasculature [22].

Currently, the clinical relevance of CEC as a predictive biomarker for anti-angiogenic therapy has only been tested in small series and results remain controversial. In our study, sunitinib treatment was associated with a significant increase of CEC during the initial 4 weeks of treatment in patients who achieved a PFS above the median, whereas patients with a PFS below the median exhibited no significant increase in CEC. These findings are supported by other studies, which also determined significant changes of early biomarkers within 4 weeks of treatment [4,23]. Prolonged treatment with sunitinib has been studied in a subgroup of patients with repeated measures only. In these patients CEC counts decline to a basal level with sustained sunitinib treatment. Therefore, we share the notion that the therapy-induced CEC elevation may represent an early marker for superior clinical response to sunitinib.

Some limitations are associated in regard to studies exploring the nature of CEC in patients. Different evaluation tools and sampling time points may lead to conflicting results. In the phase II study of pazopanib, an inhibitor of VEGFR1-3, KIT, and PDGFR, evaluation of biomarkers was included at week 12 [24]. CEC showed no correlation with tumor response. Our data supports the notion that CEC may be an early marker, which must be determined within 4 weeks of therapy initiation in order to observe biological activity. The mere detection of CEC counts obviously raises numerous questions and awaits the development of a more detailed view to CEC function and vitality, such as analyses of apoptosis or endothelial cell activation.

We also investigated the potential of circulating monocytes and sVEGFR2 as putative surrogate markers to assess sunitinib's biological activity. Monocytes are known to express VEGFR1, KIT, and PDGFR and, thus, may reflect a valuable tool to monitor target inhibition. Treatment with sunitinib resulted in a marked decrease of monocyte counts in all patients, which may suggest adequate target inhibition through sunitinib irrespective of their clinical response to treatment. As a consequence of target inhibition, a marked proportional decrease of monocytes has also been previously reported by other investigators [4]. In addition, neutrophils and monocytes have been shown to be elevated in non-responding RCC patients treated with sunitinib [25]. These studies underscore the role of monocytes in the process of response to sunitinib and warrant further studies.

Treatment with sunitinib correlated with increased VEGF and decreased sVEGFR2 in patient serum, but correlation with clinical outcome remained mainly undefined [26-28]. In breast cancer patients, only decreased sVEGFR3 and sKIT were thought to correlate with clinical outcome [13]. In RCC patients, no correlation for VEGF, sVEGFR2, sVEGFR3 and efficacy could be determined [29]. Our results support the notion that sunitinib-induced target inhibition is associated with decreased sVEGFR2 concentrations, but the kinetic modulation of sVEGFR2 levels is insufficient to predict tumor response throughout the course of therapy.

Our results underline the importance of the incorporation of biological markers in anti-angiogenic therapies in order to precisely determine the response to therapy. The increase of CEC may represent a valuable tool to predict biological response in RCC patients undergoing sunitinib treatment, but time of analysis seems to be crucial in order to detect the clinical relevance of CEC numbers. The small sample size in our analyses hampers the applicability of our results to the clinic and requires further careful validation. Clearly, there is still no single, magic predictive marker for anti-angiogenic therapy. Rather, a panel of biomarkers has to be considered in order to obtain a detailed picture of the effectiveness of the given therapy. Our findings suggest that CEC may be a valuable biomarkers to predict superior PFS response to sunitinib.

## Conclusions

Early increase of circulating endothelial cells is associated with superior PFS response to sunitinib and may serve as an early predictive biomarker. However, monocytes and sVEGFR-2 may exert pharmacodynamic changes, but fail to correlate with PFS of sunitinib treatment. Further prospective studies are needed to determine the role of soluble markers in RCC.

## Competing interests

Viktor Grünwald received consultancy and honoraria from Pfizer Oncology, Roche Pharma, GSK and Novartis Oncology. A research grant was provided by Wyeth Oncology and lecture honoriarium from Bayer. The following authors did not declare any competing interest: Gernot Beutel, Susanne Schuch-Jantsch, Christoph Reuter, Philipp Ivanyi, Arnold Ganser, and Marion Haubitz.

## Authors' contributions

VG drafted the manuscript and design of the study, collected and analyzed the clinical data and correlated with biomarker data. GB co-drafted the design of the study and coordinated specimen collection. SSJ carried out soluble biomarker tests. CR and PI collected, gathered and coordinated clinical data. AG provided general support and helped to draft the manuscript. MH planned and conducted the analyses of CEC and participated in the design of the study. All authors read and approved the manuscript.

## Pre-publication history

The pre-publication history for this paper can be accessed here:

http://www.biomedcentral.com/1471-2407/10/695/prepub
